# CypA的生物学功能及其与肺癌相关的研究进展

**DOI:** 10.3779/j.issn.1009-3419.2010.08.15

**Published:** 2010-08-20

**Authors:** 文涛 岳

**Affiliations:** 1 101149 北京，北京胸科医院综合科 General Department, Beijing Chest Hospital, Beijing 101149, China; 2 101149 北京，北京市结核病胸部肿瘤研究所细胞室 Department of Cellular and Molecular Biology, Beijing TB and Toracic Tumor Research Institute, Beijing 101149, China

**Keywords:** 亲环素A, 肺肿瘤, 环孢素A, CD147, Cyclophilin A, Lung neoplasms, Cyclosporine A, CD147

## Abstract

亲环素A（cyclophilin A, CypA）作为亲环素家族中最重要的一员，在自然界中广泛表达，发挥着肽基脯氨酰顺反异构酶（peptidyl-prolyl cis-trans isomeras, PPⅠase）活性和分子伴侣效应，辅助细胞内蛋白质的正确折叠，参与免疫抑制，介导炎性反应，平衡细胞内外胆固醇。随着对CypA认识的加深，人们逐渐意识到它与恶性肿瘤之间的密切联系。CypA最先被发现在肺癌中高表达，具有促进肺癌细胞生长、抑制凋亡、介导侵袭转移等作用，可能成为一个肺癌的早期诊断和治疗的新靶点。

## Abstract (en)

CypA is the most important member of Cyclophilins. It is a widely expressed protein in nature possess-ing PPIase and chaperone activities which help the precise folding of protein. Also, it is involved in immunosuppression, inflam-mation and the balance of cholesterol. As the understanding of CypA function has deepened, people began to realize that there might exist a relationship between CypA and cancer. Lung cancer is the first carcinoma which was found as expressing high level of CypA. CypA plays the roles in increasing proliferation, antiapoptosis, invasion and metastasis in lung cancer. The study of CypA may open a new window for the early diagnosis, prognosis and novel therapeutic drugs targeting.

肺癌已经成为对人类健康威胁最严重的恶性肿瘤，发病率和病死率逐年上升。根据卫生部公布的第三次全国居民死亡原因调查结果显示，我国肺癌的死亡率已经高达30.83/10万，比30年前上升了465%。肺癌的病因至今仍不完全明确，吸烟、遗传因素、环境污染、自身免疫状态、肺部慢性疾病等均被认为对肺癌的发病有影响。近年来，伴随着分子生物学领域的发展，人们对肺癌的发生发展有了更加深入的认识，亲环素A（Cyclophilin A, CypA）与肺癌的关系也随着分子生物学研究的发展而被揭示出来。CypA在多种恶性肿瘤中呈高表达，并且与肿瘤相关蛋白的合成、促进肿瘤细胞生长信号的传递、抑制凋亡、细胞运动调节蛋白、转录因子等均有密切的关系。因此，作为一个潜在的早期诊断和靶向治疗因子，CypA与肺癌的生物学关系的研究或将成为一个有前景的方向。本文将对CypA与肺癌相关的研究进展做一综述。

## 1 CypA的发现与结构特点

### 1.1 CypA的发现

CypA是亲环素家族（Cyclophilins, Cyps）中的重要一员，也是最早被发现的亲环素。亲环素家族最先被认为是环胞素A（Cyclosporine A, CsA）的胞内结合蛋白，其由一组具有肽基脯氨酰顺反异构酶活性的蛋白质所组成，在进化上有高度的保守性，广泛表达于原核生物和真核生物中。亲环素家族有16个成员，其中常见的在人体内表达的有6个，包括CypA、CypB、CypC、CypD、CypE（Cyp33）和Cyp40。CypA是最早被提纯的亲环素家族成员，早在1984年Handschumacher等^[1]^即利用高效液相色谱法（high performance liquid chromatography, HPLC）从小牛胸腺中提取到了CypA，并为其命名。迄今为止，CypA仍是研究最广泛的亲环素。

### 1.2 CypA的分子结构特点

CypA由一条含有165个氨基酸的多肽单链构成，分子量约为18 kDa。20世纪90年代初，科学家利用X射线晶体衍射技术（X-ray crystallography）和核磁共振（nuclear magnetic reson, NMR）技术对CypA的蛋白质结构进行了剖析。研究^[2, 3]^发现，在蛋白质二级结构水平，除了少量的α-螺旋、β-转角及无规卷曲外，CypA主要由β-折叠组成。其中最具结构特征的是8条反向平行的β-折叠片段，与两端的α-螺旋结构共同构成了CypA的桶状疏水性中心。此中心内包含了影响CypA生物学活性的三个重要的疏水性残基，它们是Arg55、Gln63和His126，CypA与大多数配体作用时氢键的形成均与这三个残基密切相关。

## 2 CypA的表达与生物学功能

### 2.1 CypA的表达与分布特点

人类编码CypA的基因位于染色体7p13位点，长2 276 bp，内含5个外显子区域^[4]^。CypA在原核生物和真核生物中均广泛表达。由于CypA高度的进化保守性，使得它在各种生物和组织中的表达差异很小^[5]^。在细胞水平上，CypA主要定位于胞浆，也部分表达在高尔基体和胞核内。某些细胞，如血管平滑肌细胞在受到氧化应激或者巨噬细胞在受到脂多糖刺激时，它们还可以通过囊泡运输的方式将CypA分泌到胞外，发挥类似细胞因子的功能^[6]^。CypA在人体中的各种正常组织均有表达，占细胞内蛋白表达量的0.1%^[7]^。其中表达量最高的是大脑皮质，约为2.8 μg/mg蛋白。在正常肺组织中CypA的表达量约为0.8 μg/mg蛋白。免疫组化显示CypA在肾脏的近端小管上皮较其它组织呈高表达，这恰好与CsA的肾毒性作用位点相关^[8]^。

### 2.2 CypA的生物学功能

#### 2.2.1 肽基脯氨酰顺反异构酶（peptidyl-prolyl cis-trans isomeras, PPIase）活性

在人体中，CypA发挥着多种生物学功能，其中最早引起人们注意且最重要的即为CypA的PPIase活性。目前认为PPIase对于含有脯氨酸的蛋白质的活性空间结构的形成具有催化作用，是关键限速酶之一^[9]^。在折叠起始过程中，催化蛋白质在顺式和反式结构之间的转化以及维持蛋白质多聚体的构象均有赖于CypA的PPIase活性^[10]^。同时，也有研究^[11]^发现CypA可起到分子伴侣的作用，在细胞遇到诸如热损伤或者紫外线照射等不良刺激时，CypA可参与损伤蛋白的修复。CypA的PPIase活性促进了蛋白质的合成和功能的发挥，为细胞的增殖提供了物质保证^[12]^。基于CypA的PPIase活性，人们还发现在氧化应激条件下，血管平滑肌细胞可以分泌CypA，其通过ROS介质激活ERK1/2，促进血管平滑肌细胞的增殖，并且能够趋化炎症细胞和血管内皮细胞至损伤部位，参与血管的重构，提示CypA与血管损伤疾病，如高血压、动脉粥样硬化、肿瘤血管新生的发病机制有关^[13]^。

#### 2.2.2 介导CsA参与的免疫抑制

CsA是一种在临床上广泛应用的免疫抑制剂，多用于抑制器官移植后的免疫排斥反应。CypA是CsA最重要的也是发现最早的胞内受体^[14]^。CsA可以与CypA的疏水性活性中心相结合形成CypA:CsA二元复合物，发挥促进T细胞凋亡、阻断信号传导、抑制T细胞活化，从而达到抑制免疫的作用。当CsA进入T细胞后，可以激活CypA的核酸酶活性，降解DNA，参与T细胞的凋亡过程^[15]^。CypA:CsA二元复合物可以阻断MAPK信号通路中的p38、MAPK和JNK的活化，抑制*IL-2*、*IL-4*等基因的转录激活过程，致使T细胞停留在G_0_期，阻碍T细胞的增殖。同时，CypA:CsA二元复合物还可以抑制钙调神经素（calcineurin, CaN）的酶活性，阻断其去磷酸化活化T细胞核因子（nuclear factor of activated T cell, NF-ATc），下调淋巴因子的表达，抑制T细胞的活化，产生免疫抑制的功能^[16]^。实验^[17]^证明，在CypA基因缺陷的小鼠体中，CsA无法发挥免疫抑制作用。因此，人们逐渐认识到CypA与某些免疫缺陷疾病密切相关，如艾滋病、系统性红斑狼疮等。其中研究最为深入的是CypA与艾滋病发病机理的关系。CypA能特异性结合HⅣ-1（human immunodeficiency virus type 1）病毒的gag蛋白，在其进入宿主细胞后，帮助病毒核心在脱衣壳过程中解装配^[18]^。这一发现已经被应用于抗艾滋病药物的研究中^[19]^。

#### 2.2.3 参与胆固醇的代谢

CypA在细胞膜上可与Caveolin-1形成复合体，参与细胞质膜微囊（Caveolae）的合成，介导细胞内外胆固醇的转运，维持细胞内外胆固醇的平衡。在细胞内CypA则与Caveolin-1、CyP40和HSP56形成复合物，充当细胞质内的胆固醇运输载体^[20]^。细胞膜上介导Caveolae转运胆固醇的关键蛋白是Caveolin-1，CypA的内部含有Caveolin-1结合区和特异的热休克蛋白结合区，可以通过改变疏水区两端的脯氨酸残基的顺-反式结构，加速蛋白折叠，使Caveolin-1在空间构象上更有利于胆固醇的结合而影响胆固醇的代谢^[21]^。由此可以推断CypA与脂代谢疾病的发生有关。在动脉粥样硬化动物模型上，Jin等^[22]^发现巨噬细胞中的清道夫受体可以调控CsA的浓度，提示作为CsA胞内受体的CypA可以对动脉粥样硬化的发病产生影响。

#### 2.2.4 参与炎症反应

1997年，Billich等^[23]^在26例类风湿关节炎（rheumatoid arthritis, RA）患者的膝关节滑液里检测到CypA，同时发现CypA的含量与关节滑膜中的中性粒细胞的数量呈正比。近期有研究^[24]^发现，RA患者血清和滑液中的CypA表达量与RA的发病严重程度正相关，由此可以认为CypA与炎症反应有关联，可在炎症反应中发挥类似细胞因子的功能。实验^[25]^证明CypA在炎症反应中发挥功能主要依赖与受体CD147分子的结合。免疫球蛋白CD147在CypA激活ERK的信号级联反应中发挥关键作用。CypA与CD147的相互作用可以趋化单核细胞、中性粒细胞和嗜酸粒细胞等炎症反应细胞至炎症发生部位^[26]^。在应用抗CD147的抗体后，CypA的趋化效应受到明显的抑制，炎症反应也大幅下降^[27]^。

## 3 CypA与肺癌

### 3.1 CypA在肺癌中的表达

第一次揭示CypA在恶性肿瘤中的表达情况是在其被命名20年后。2003年，Campa等^[28]^应用“基体辅助激光解吸电离飞行时间质谱（MALDI-TOF MS）”技术检测了10例非小细胞肺癌组织以及相配对的10例正常肺组织，发现了CypA的表达差异非常明显。肺癌组织中的CypA含量是正常组织中的7倍，Western blot、免疫印记和免疫组化方法也同样支持这一结果。此后Campa等^[29]^又比较了234例组织学上不同分级的原发性非小细胞肺癌标本的组织芯片免疫组化（tissue microarray immunohistochemistry, TMA-IHC），结果也证明CypA在肺癌组织中表达显著升高。在表达CypA的组织中有25%呈强阳性，37%呈阳性，38%呈弱阳性。在A549、H1299、H446、SPC-A1等5种肺癌细胞系中H446的CypA的表达量最高，是人肺成纤维细胞系Hs8881u的6倍。CypA的配体CD147在这5种细胞系中的表达量与CypA呈正比^[30]^。近些年来，应用2D电泳、质谱分析、MALDI-TOF MS、Realtime PCR、Western blot、免疫组化等方法，人们先后发现了CypA在胰腺癌、肝癌、乳腺癌、子宫内膜癌、结直肠癌以及其它恶性肿瘤中的表达均升高^[31]^。

### 3.2 CypA对肺癌生物学性状的影响

CypA在肺癌组织中的高表达引起了科学家的关注，促进了人们对CypA对肺癌生物学性状方面作用的探讨。Campa等^[28]^在利用基因干扰（RNA interference, RNAi）技术抑制了ADLC-5M2和LC-103H两种非小细胞肺癌细胞系的CypA表达后，将细胞系接种于裸鼠，1个月后发现两种经过RNAi后的肺癌细胞的活体成瘤体积相对于正常肺癌细胞分别下降了72%和87%，这一结果表明下调CypA的表达可以明显地抑制肺癌细胞的生长。同时，比较转入了过表达*CypA*基因和转入空载基因载体的小气道上皮细胞在裸鼠体内的生长情况后，发现CypA的过表达能够显著促进小气道上皮细胞在活体中的生长^[32]^。另外，研究^[30]^证实外源性的CypA可以促进小细胞癌细胞系的生长。异常血管新生也是恶性肿瘤的特征性表现之一，然而抑制CypA表达后并未对肺癌的血管新生产生显著影响。但是，由于先前有报道^[33]^称外源性的CypA可以促进人脐静脉内皮细胞的增殖、转移和血管生成，而且在低氧和炎症的情况下CypA还可被内皮细胞和血管平滑肌细胞分泌至细胞外发挥功能^[34]^，因此，不排除是检测方法和范围的局限影响了CypA对于血管生成关系的判断。除此之外还发现，抑制CypA对肺癌细胞的凋亡有很大的影响。利用原位末端标记法（terminal deoxyribonucleotidyl transferase-mediated dUTP nick-end labeling, TUNEL）检测出RNAi后的肺癌细胞系的凋亡数量明显上升，提示CypA具有增强肺癌细胞抗凋亡的能力，可能起着保证肿瘤细胞存活功能。抑制CypA还会对肺癌细胞的代谢产生影响，可以使癌细胞对葡萄糖的吸收能力减弱，代谢活动下调^[32]^。

### 3.3 CypA对肺癌作用的分子机制

CypA通过多种机制发挥它对肿瘤的影响。针对肺癌方面，主要表现在促进肺癌细胞的增殖，这与CypA的PPIase活性和CD147分子密切相关。CypA的PPIase活性和分子伴侣效应可以催化合成肺癌细胞生存生长所必须的蛋白，提高肿瘤细胞抵抗各种不良刺激的能力。在H446细胞系中添加拮抗PPIase活性的物质，如CsA等，CypA对H446细胞的促生长效应立刻受到抑制。CypA对H446细胞生长的促进还可能是通过其对ERK1/2信号通路的激活来实现的，CypA首先与CD147结合后再作用于ERK1/2信号通路，拮抗CD147同样能抑制CypA对细胞生长的促进^[30]^。最近有研究^[27]^发现，CypA可以通过CD147促进RA患者的炎症细胞分泌金属蛋白酶-9（matrix metalloproteinase-9, MMP-9）。众所周知，MMP-9在恶性肿瘤中可以促进肿瘤侵袭转移的能力，刺激VEGF的分泌，参与多药耐药^[35]^。虽然尚无完整的实验证据，但是不难想象CypA可以通过CD147的协助，刺激MMP-9在肺癌的发生发展中发挥作用。已有研究证实了CypA对于小细胞肺癌细胞的抗凋亡能力，有人提出CypA可以抵抗缺氧和抗癌药物对于肿瘤的促凋亡作用，但是其分子机制尚不明了，多数人认为这与减少活性氧物质的威胁和线粒体通透性转换孔的调节有关^[34]^。有关证实CypA参与肿瘤多药耐药的研究^[36]^认为，高表达CypA的肿瘤细胞对阿霉素和长春新碱的耐药水平均有提高。近期有研究把CypA作为肿瘤治疗的新靶点，希望通过对它的抑制延缓肿瘤的生长。

### 3.4 CypA与肺癌的早期诊断

Howard对234例肺癌组织的CypA表达情况做统计学分析发现CypA的表达高低与组织病理分级和病人预后之间无显著的相关性，作为预后指标的可能性不大。那么CypA在不同分期和分级的非小细胞肺癌组织中的高表达究竟能说明什么问题呢？进一步分析后研究^[29]^认为CypA可能是在肺癌早期癌细胞向恶性转化时起作用，而在后期的分化和侵袭转移中可能不起显著作用。这点恰好可以用CypA的分子生物学作用机制解释：在肺癌形成的早期，CypA的表达明显升高，充分发挥PPIase活性而加速癌组织相关蛋白的折叠和合成，即可为肿瘤的恶性生长提供物质保证。作为一种主要存在于胞质中的蛋白，在应激条件下CypA可以被分泌到细胞外发挥作用。Suzuki等^[6]^发现在应用超氧化物激活剂刺激血管平滑肌细胞后采用Western blot的方法可检测出CypA在培养基中的表达。Brune等^[37]^在重症败血症患者的血清中也检测到了高表达的CypA。因此虽然还未出现有关CypA在肺癌患者血清中表达含量的数据，但是也不排除可以在肺癌患者血清标本中检测出CypA，这可以大大地提高CypA作为肺癌诊断标志物应用的可行性。另外，不同个体之间血清蛋白含量的影响因素较多，就肺癌而言，胸水通常是由肿瘤直接刺激产生，与疾病发展关系密切，特异性更高，因此胸水中是否表达CypA以及表达量高低也是值得探讨的问题。虽然暂无比较诊断肺癌组织中CypA方法敏感性和特异性的实验，但考虑到CypA是一种广泛表达的蛋白，在正常组织中含量也较高，可以推断出CypA应该是一个敏感性高而特异性较低的早期诊断指标。

## 4 结语与展望

作为一个表达广泛、功能多样的蛋白质明星，CypA一直吸引着科学家们的眼球。PPIase活性、免疫抑制作用、介导炎症反应等诸多功能的发现以及蛋白质组学和分子生物学技术上的革新和进步，自然而然地将研究CypA的目光瞄准了恶性肿瘤。最早在肺癌中发现CypA的高表达后，人们就意识到它会与肺癌的发生发展有着密切的关系，随后发现它在肺癌中的促生长、抗凋亡、侵袭转移和多药耐药方面的作用也证实了人们的假设^[38]^。当然，关于CypA还有很多问题需要我们去解决：CypA在肺部良恶性病变中表达数据尚不全面，表达量范围尚未界定；CypA的促肺癌细胞生长的分子机制尚不明确；CypA的PPIase活性在肺癌中发挥作用的途径尚不全面；CypA如何抗肺癌细胞凋亡；CypA是否有促肿瘤新生血管形成的作用；CypA的抑制能否帮助抗肿瘤药物更好地发挥作用等等均亟待更加深入的研究。作为一个潜在的肺癌早期诊断标志物和治疗靶点，相信有关CypA的研究一定能为人们认识和解决肺癌难题开启新的篇章。
